# A Mixed Methods Investigation of Stress and Wellbeing Factors Contributing to Burnout and Job Satisfaction in a Specialist Small Animal Hospital

**DOI:** 10.3389/fvets.2022.942778

**Published:** 2022-06-24

**Authors:** Claire E. Ashton-James, Amy G. McNeilage

**Affiliations:** Sydney Medical School, Faculty of Medicine and Health, The University of Sydney, Sydney, NSW, Australia

**Keywords:** specialist veterinary hospital, small animal, stress, wellbeing, veterinary care workers, qualitative, MBI-GS

## Abstract

Occupational burnout is a critical issue affecting the welfare of veterinary care providers, their patients, and the sustainability of veterinary healthcare organizations. The current research aimed to evaluate the prevalence of and factors contributing to stress, wellbeing, burnout symptoms and job satisfaction among clinical and non-clinical staff at a large specialist small animal hospital in Australia. Participants completed an anonymous online survey including self-report measures of job satisfaction and symptoms of burnout, and open-text response questions probing sources of stress and wellbeing. Subsequently, participants rated how frequently they experienced commonly reported sources of veterinary stress, and a series of focus groups were conducted with clinical and non-clinical teams. The survey was completed by 249 participants (overall response rate = 70%; 67.1% “clinical;” 17.3% “non-clinical;” 5.6% “other”). Five focus groups (including 38 of the survey participants) were subsequently conducted with groups of clinical and non-clinical staff. The majority of respondents (80.7%) reported being satisfied, very satisfied, or extremely satisfied with their job. At the same time, 57.7% of respondents exceeded the threshold for burnout on at least one burnout dimension, with 48.1% reporting high levels of emotional exhaustion, 30.2% reported high levels of cynicism, and 16.3% reporting low levels of professional efficacy. Open text responses and focus group transcripts revealed three common sources of stress and wellbeing. Stressors included communication (conflict within teams), clients (dealing with client emotions and expectations), and heavy caseload. Wellbeing was enhanced by people (team cohesion, respect for colleagues), practice (variety, autonomy, challenge), and purpose (meaningful work and impact). Overall, for both clinical and non-clinical survey respondents, “heavy workload” was rated as the most frequent source of stress. Despite high levels of job satisfaction, approximately two thirds of respondents reported at least one symptom of burnout. Convergent results from the survey and focus groups indicated that strong relationships with colleagues and the intrinsic meaningfulness of the work were key sources of wellbeing and job satisfaction. On the other hand, challenging workplace interactions with colleagues and clients, as well as heavy workload, were identified as key stressors contributing to burnout symptoms.

## Introduction

Animal care workers typically report high levels of meaning at work and job satisfaction (1–3). At the same time, research indicates that the prevalence of burnout symptoms is significantly higher among veterinary care teams, including veterinarians, veterinary students, and veterinary nurses/technicians than in the general population (4–7). Indeed, veterinary teams may be more susceptible to burnout than human medical professionals (6, 8).

Occupational burnout is a psychological syndrome comprising of emotional exhaustion (extreme fatigue resulting in a feeling of emotional “numbness”), depersonalization or cynicism (disengagement and reduced concern for colleagues and patients) and a low sense of personal accomplishment (perceived lack of control over the quality of one's work or outcomes) (9). High levels of burnout may have potentially serious consequences for individual sufferers, their patients, and veterinary care organizations. Higher levels of burnout in veterinary care providers are associated with higher employee absenteeism and attrition in the workforce (10–12), causing professional shortages and significantly affecting patient access to care (13). Practitioner stress and fatigue is a common cause of error in veterinary practice (14). Veterinary carers experiencing high levels of occupational burnout have increased risk of insomnia and depression, substance abuse disorders, and numerous chronic health conditions including coronary heart disease, type 2 diabetes, and respiratory problems (15). Most concerning, burnout in veterinary care providers is associated with a higher risk of suicidal ideation. It is estimated that suicide rates are two to four times higher among veterinarians than the general population, and up to two times higher than human healthcare workers (16, 17).

Despite evidence for the prevalence of burnout in veterinary medicine, there remains a dearth of research examining strategies to address this problem. A 2018 systematic review found only four original research articles evaluating therapeutic interventions to mitigate symptoms of burnout (occupational stress and compassion fatigue) in animal care workers (18). The authors concluded that “this small number, combined with the variability in design and outcome measures of the articles, made best practice recommendations on the basis of this review difficult.” The authors recommended that organizations seeking to implement interventions should borrow from research conducted in other areas until a strong research base is established. However, research conducted across industries indicates that sources of stress and burnout, and contributors to wellbeing and job satisfaction are idiosyncratic across industries and professions (19). Indeed, factors contributing to burnout may vary from organization to organization (20). Hence, a targeted approach is recommended, whereby interventions are developed based on an understanding of the most salient contributors to burnout in each organization.

The current research study was designed with the aim of understanding factors contributing to burnout and job satisfaction in a large specialist small animal hospital in Australia. While previous research has tended to focus entirely on sources of stress that may be precipitating burnout among veterinary care providers, the current research also seeks to understand sources of wellbeing among veterinary care workers. Feelings of wellbeing (positive emotion, meaning, purpose, engagement and connectedness) (21) contribute to job satisfaction and are associated with retention of staff, and work performance (22–25). In addition, feelings of wellbeing may increase individuals' resilience and ability to cope with stress, thereby helping to mitigate burnout (24, 26–28). Hence, in addition to exploring sources of stress that may be contributing to burnout, we aimed to uncover sources of wellbeing that may be contributing to job satisfaction in the context of an Australian specialist small animal hospital.

Previous research into burnout and job satisfaction in veterinary care organizations has tended to focus exclusively on the experience of clinical staff. However, burnout also effects non-clinical professionals (29), who play a critical role in the delivery of service to animals and their carers. Non-clinical staff (e.g., managers, financial officers, human resource professionals, client services, operations team, and marketing) facilitate patient care by scheduling appointments and procedures, staffing clinical teams, procurement of products, medications, and equipment, marketing of services, arranging client payments, and management of business operations. Despite the potential for burnout among non-clinical staff to impact on the wellbeing and performance of clinical staff, the prevalence of burnout and job satisfaction among non-clinical staff in veterinary care organizations is unknown. Hence, the current research aims to understand stress and wellbeing factors contributing to burnout and job satisfaction in non-clinical as well as clinical staff.

## Methods

The study used mixed methods (i.e., quantitative and qualitative; survey and focus groups). Ethical approval for the study was granted by the Human Research Ethics Committee of the Northern Sydney Local Health District (reference: 2020/ETH00347).

### Setting and Participants

The study was conducted in a large specialist veterinary hospital in Australia that employs 357 staff in total, including 121 specialist and non-specialist veterinarians, 160 veterinary nurses/technicians and animal attendants, 33 client services staff (reception and contact center), 31 support services staff (finance, IT, procurement, operations, marketing, and human resources), as well as 12 other leadership staff. All staff were eligible to participate in the study, which was conducted between July and October 2021. Although the study was conducted during the global COVID-19 pandemic, the majority of staff worked onsite (i.e., not from home) as they were classified as “essential workers.”

### Procedure

An anonymous online survey was hosted on Qualtrics (Qualtrics, Provo, UT) and a secure survey link was sent to every staff member by the head of Human Resources. Staff were encouraged to participate in the survey to enable the hospital to develop tailored interventions to support wellbeing in the workplace. The survey data collection was managed by researchers who were external to the organization (CA-J and AM). The survey was initially available for participation for a period of 4 weeks. Researchers CA-J and AM analyzed the survey data, including quantitative data and open-ended survey responses, in order to identify specific “high risk” and “low risk” teams to participate in focus groups. High-risk groups were those with high levels of burnout symptoms (i.e., above established cut-offs) and low-risk groups were those with low levels of burnout symptoms (i.e., below established cut-offs) (9).

Five focus groups were conducted with veterinarians (*n* = 6), nurses (*n* = 7), nurse leaders (*n* = 6), frontline and operational leaders (*n* = 9), and staff from client services and support services (*n* = 10). The focus groups were designed to evaluate the reliability of survey results as well as to gain a deeper understanding of the sources of wellbeing and stress identified among high-risk and low-risk groups. Focus groups were conducted online (using Zoom meeting software) and were facilitated and recorded by the principal investigator (CA-J). Focus groups were semi-structured, starting with a brief overview of the survey purpose and results, followed by a series of open questions designed to elicit each groups' reflections on the results (e.g., “What do these results mean to you?” “Are there results that resonate with you?” “Do any results surprise you?”). As participants reflected on the results, the facilitator used probing questions (e.g., “Tell me more about that,” “why do you think that is?” “How does that affect you, if at all?”) were used to unearth common sources of stress or wellbeing. Finally, when time permitted, focus group participants were encouraged to express their thoughts about how factors contributing to stress may be addressed. Participation was voluntary and limited to those who were available at the time.

### Survey Measures

Measures were designed to be brief in consideration that heavy workloads are common in the surveyed population (i.e., for feasibility).

Job satisfaction was measured using a single item (“On the whole, how satisfied are you with your job?”) scored on a 7-point Likert scale, with response options ranging from “1 = extremely dissatisfied” to “7 = extremely satisfied.” Single-item questions are a valid and reliable means of measuring global job satisfaction (30, 31).

Symptoms of burnout were measured using the 16-item Maslach Burnout Inventory General Survey (MBI-GS) which consists of subscales for the three dimensions of burnout: emotional exhaustion (EE), cynicism (CY), and low professional efficacy (PE). Items are scored on a 7-point Likert scale, with response options ranging from “0 = never” to “6 = every day” (9). The emotional exhaustion subscale includes five items (e.g., “I feel emotionally drained from my work;” “Working all day is really a strain for me”), the cynicism subscale includes five items (e.g., “I have become more cynical about whether my work contributes anything;” “I have become less enthusiastic about my work”), and the professional efficacy subscale includes six items (e.g., “In my opinion, I am good at my job;” “I feel I'm making an effective contribution to what this organization does”). The MBI-GS has been reported to have acceptable reliability with Cronbach's alphas of 0.91 for the emotional exhaustion subscale, 0.86 for the cynicism subscale, and 0.76 for the professional efficacy subscale (32). Higher scores on the emotional exhaustion and cynicism subscales and lower scores on the professional efficacy subscale indicate greater levels of burnout. The following thresholds were used to establish high levels of burnout symptoms: ≥3.2 for EE, ≥2.6 for CY, and ≤3.8 for PE (9).

Sources of stress and wellbeing at work were probed with two open-text questions: “What, if anything, do you enjoy about working at [veterinary hospital]?” (Q1) and “What, if anything, do you find stressful about working at [veterinary hospital]?” (Q2).

Exposure to common sources of stress in veterinary care was evaluated by asking participants to rate the extent to which a variety of factors (see **Table 4**) contributed to their experience of stress at work on a 5-point scale ranging from “1 = not at all” to “5 = a great deal.” Potential sources of stress were derived from a review of research into factors contributing to stress and burnout in non-human animal (veterinary) carers (6, 13, 33–45).

Demographic questions included participant gender (female, male, other, prefer to self-describe, prefer not to say), position (specialist vet, non-specialist veterinarian, resident / intern, veterinary nurse, trainee nurse / animal attendant, client services / contact center, support services, leadership team, prefer not to say, other) years working in the veterinary care sector (<1 year, 1–5, 6–10, and more than 10 years), and years at current workplace (<1 year, 1–5, 6–10, and more than 10 years).

### Data Analysis

Survey data were analyzed using JASP (JASP Team, 2020; version 0.14.1). JASP is a free and open-source statistical software program supported by the Psychological Methods Group at the University of Amsterdam. Descriptive statistics, means, and standard deviations were used to describe job satisfaction and symptoms of burnout. To explore whether job satisfaction and burnout symptoms differed between clinical and non-clinical staff, independent samples *t*-tests were performed. Where the homogeneity of variance assumption was violated, indicated by a significant Levene's test (*p* < 0.05), Welch's *t*-test was used. The threshold for significance was set at *p* < 0.05/4 = 0.0123 for this series of *t*-tests using Bonferroni correction to account for multiple comparisons. Correlations were calculated to explore the associations between symptoms of burnout, job satisfaction, and prevalence of common stressors. Because 82 bivariate correlations were tested, the threshold for significance was set at *p* < 0.05/82 = 0.0006 using Bonferroni correction for multiple comparisons. Multiple linear regression was conducted with emotional exhaustion, cynicism, and professional efficacy simultaneously entered into the model as predictors to identify the symptoms of burnout most strongly associated with job satisfaction. Prior to running the analysis, assumptions of regression were checked using the Durbin-Watson statistic and the variance inflation factor (VIF), as well normal probability and residual plots.

Qualitative data from open-ended survey questions and focus groups was analyzed by two researchers (CA-J and AM) who followed Braun and Clarke's stepwise procedure for thematic analysis (46). Thematic analysis was chosen as the analytic method as our goal was to gain a rich understanding of the similarities and differences in participants' experiences of stress and wellbeing in the workplace. First, both researchers (CA-J and AM) read all survey responses and all focus group transcripts several times. Once the researchers had familiarized themselves with the dataset, one (CA-J) inductively coded the survey responses while the other (AM) inductively coded the focus group transcripts. Themes were generated through an iterative and collaborative process of grouping descriptive codes into more meaningful higher-order categories. The researchers reached consensus on the final thematic structure, agreeing it accurately captured the essence of the dataset. Exemplar quotes were selected from both survey responses and focus group transcripts to demonstrate the trustworthiness of the agreed upon themes.

### Reflexivity

No one on the research team had prior experience conducting research or professional work in the veterinary industry and therefore they may have had fewer (or less specific) expectations of the results. At the same time, the first author (CA-J) has researched burnout among medical professionals and her interpretation of the data in this study may have been influenced by themes identified in previous studies. It should also be noted that the first author (CA-J) is a social psychologist and thus may have prioritized interpersonal themes. The second author (AM) is a research assistant studying psychology. Neither author had a prior relationship with any participant.

## Results

Of 357 eligible staff, 249 answered at least one survey question and were included in the analysis where possible (response rate = 70%). Two-hundred and 26 respondents (63.3%) completed all demographic questions, while 249 (69.7%), 244 (68.3%), and 230 (64.4%) completed sections on job satisfaction, symptoms of burnout, and sources of stress, respectively. The open-ended questions were answered by 231 respondents (64.7%). Positions were classified by the authors as either clinical or non-clinical, with the majority working primarily in clinical (e.g., veterinary, nursing) roles (67.1%; *n* = 167) and the remaining working primarily in non-clinical (operations, management, client services) roles (17.3%; *n* = 43). Other participant characteristics including gender, position description, and years of experience are summarized in Table 1. Of the eight respondents who classified their position description as “other,” three reported they were radiographers, two reported they were rehab therapists, and three did not specify.

**Table 1:** Participant characteristics (*N* = 249).

**Characteristic**		***n***	**%**
Gender			
	Female	174	69.9
	Male	36	14.5
	Other	2	0.8
	Prefer not to say	16	6.4
	Unspecified	21	8.4
Role			
	Clinical	167	67.1
	Non-clinical	43	17.3
	Unspecified	39	15.7
Position			
	Specialist veterinarian	30	12.0
	Non-specialist veterinarian	11	4.4
	Resident/intern	27	10.8
	Veterinary nurse	77	30.9
	Trainee nurse/animal attendant	17	6.8
	Client services	18	7.2
	Support services	18	7.2
	Leadership	7	2.8
	Other	8	3.2
	Prefer not to say	15	6.0
	Unspecified	21	8.4
Years working in veterinary care sector			
	<1 year	28	11.2
	1–5 years	81	32.5
	6–10 years	44	17.7
	More than 10 years	73	29.3
	Unspecified	23	9.2
Years at current workplace			
	<1 year	83	33.3
	1–5 years	99	39.8
	6–10 years	28	11.2
	More than 10 years	16	6.4
	Unspecified	23	9.2

### Job Satisfaction

The overall mean for job satisfaction was 5.10 (*SD* = 1.21, range = 1–7) and the median was 5 (i.e., “satisfied”). The majority of respondents (*n* = 201; 80.7%) reported they were either “satisfied” (*n* = 106), “very satisfied” (*n* = 75), or “extremely satisfied” (*n* = 20) with their job, while 48 (19.3%) reported being less than satisfied, that is, “neither dissatisfied nor satisfied” (*n* = 22), “dissatisfied” (*n* = 13), “very dissatisfied” (*n* = 10), or “extremely dissatisfied” (*n* = 3). Level of job satisfaction is summarized by role and position in Table 2.

**Table 2:** Job satisfaction by role and position.

		**Frequency of job satisfaction ratings (%)**							***M (SD)***
		**Extremely dissatisfied (1)**	**Very dissatisfied (2)**	**Dissatisfied (3)**	**Neither dissatisfied not satisfied (4)**	**Satisfied (5)**	**Very satisfied (6)**	**Extremely satisfied (7)**	
Role									
	Clinical	1 (0.60)	5 (2.99)	11 (6.59)	13 (7.78)	77 (46.11)	49 (29.34)	11 (6.59)	5.10 (1.12)
	Non-clinical	1 (2.33)	4 (9.30)	2 (4.65)	4 (9.30)	9 (20.93)	17 (39.54)	6 (13.95)	5.12 (1.58)
Position									
	Specialist veterinarian	0 (0)	0 (0)	0 (0)	4 (13.33)	16 (53.33)	8 (26.67)	2 (6.67)	5.27 (0.79)
	Non-specialist veterinarian	0 (0)	0 (0)	1 (9.01)	0 (0)	6 (54.55)	4 (36.36)	0 (0)	5.18 (0.87)
	Resident/ intern	0 (0)	0 (0)	0 (0)	2 (7.41)	12 (44.44)	12 (44.44)	1 (3.70)	5.44 (0.70)
	Veterinary nurse	1 (1.30)	3 (3.90)	10 (12.99)	6 (7.79)	39 (50.65)	13 (16.88)	5 (6.49)	4.79 (1.25)
	Trainee nurse/ animal attendant	0 (0)	1 (5.88)	0 (0)	1 (5.88)	3 (17.65)	9 (52.94)	3 (17.65)	5.65 (1.22)
	Client services	0 (0)	0 (0)	2 (11.11)	1 (5.56)	1 (5.56)	9 (50.00)	5 (27.78)	5.78 (1.26)
	Support services	1 (5.56)	3 (16.67)	0 (0)	3 (16.67)	6 (33.33)	5 (27.78)	0 (0)	4.39 (1.61)
	Leadership	0 (0)	1 (14.29)	0 (0)	0 (0)	2 (28.57)	3 (42.86)	1 (14.29)	5.28 (1.60)
	Other	1 (12.50)	1 (12.50)	0 (0)	0 (0)	2 (25.00)	3 (37.50)	1 (12.50)	4.75 (2.12)
	Prefer not to say	0 (0)	1 (6.25)	0 (0)	2 (12.5)	8 (50.00)	4 (25.00)	1 (6.25)	5.06 (1.12)

### Symptoms of Burnout

The emotional exhaustion, cynicism, and professional efficacy subscales were completed by 244, 239, and 239 of the 249 participants, respectively. In this sample, Cronbach's alpha was 0.92 for the emotional exhaustion subscale, 0.84 for the cynicism subscale, and 0.79 for the professional efficacy subscale. The overall means for symptoms of burnout were 2.99 for emotional exhaustion (*SD* = 1.50), 1.98 for cynicism (*SD* = 1.48), and 4.74 for professional efficacy (*SD* = 0.97). Table 3 shows the means and standard deviations for each of the burnout symptoms according to demographic groupings.

**Table 3:** Symptoms of burnout according to demographic characteristics.

**Demographic characteristic**		**Symptom of burnout, mean (SD)**		
		**Emotional exhaustion**	**Cynicism**	**Professional efficacy**
Gender				
	Female	3.07 (1.51)	1.93 (1.51)	4.80 (0.94)
	Male	2.76 (1.37)	1.86 (1.35)	4.87 (0.83)
	Other	4.20 (2.55)*	2.70 (1.27)*	2.08 (0.12)*
	Prefer not to say	3.09 (1.72)	2.73 (1.77)*	4.49 (1.08)
Role				
	Clinical	3.20 (1.46)*	2.04 (1.52)	4.71 (0.97)
	Non-clinical	2.54 (1.49)	1.61 (1.43)	5.03 (0.85)
Position				
	Specialist veterinarian	2.87 (1.40)	1.76 (1.34)	4.67 (0.96)
	Non-specialist veterinarian	3.51 (1.36)*	1.71 (1.15)	5.02 (0.52)
	Resident/intern	3.29 (1.10)*	1.70 (1.16)	4.61 (0.94)
	Veterinary nurse	3.52 (1.35)*	2.43 (1.59)	4.68 (1.03)
	Trainee nurse/animal attendant	2.62 (1.99)	1.73 (2.02)	4.88 (1.10)
	Client services	2.08 (1.57)	1.28 (1.29)	5.07 (0.90)
	Support services	2.91 (1.43)	2.18 (1.61)	4.92 (0.82)
	Leadership	2.77 (1.26)	1.00 (0.70)	5.21 (0.90)
	Other	1.85 (2.02)	1.78 (1.38)	4.27 (1.14)
	Prefer not to say	2.55 (1.57)	2.21 (1.48)	4.77 (0.86)
Years working in veterinary care sector				
	<1 year	2.14 (1.64)	1.41 (1.42)	4.84 (1.11)
	1–5 years	3.15 (1.54)	2.15 (1.63)	4.70 (0.95)
	6–10 years	3.56 (1.33)*	2.15 (1.31)	4.54 (1.00)
	More than 10 years	2.90 (1.40)	1.93 (1.49)	4.90 (0.88)
Years at current workplace				
	<1 year	2.63 (1.56)	1.73 (1.54)	4.75 (1.13)
	1–5 years	3.27 (1.46)*	2.15 (1.50)	4.78 (0.78)
	6–10 years	3.51 (1.44)*	2.47 (1.47)	4.47 (1.01)
	More than 10 years	2.74 (1.33)	1.51 (1.19)	5.08 (0.92)

*High levels of emotional exhaustion = mean ≥3.2, high levels of cynicism = mean ≥2.6, for CY, and low levels of professional efficacy = mean ≤3.8. Group means exceeding these thresholds are marked with asterisks*.

**Figure 1:**
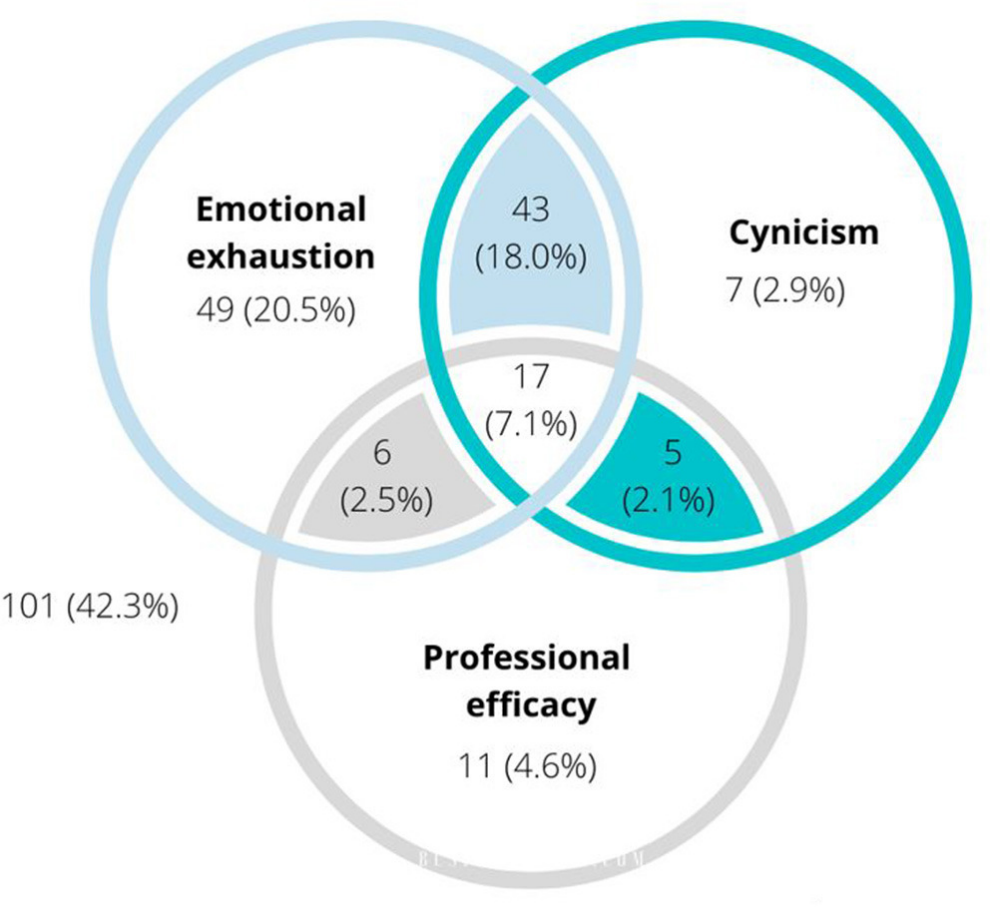
Venn diagram indicating the proportion of MBI respondents (*n* = 239) reporting symptoms of burnout (emotional exhaustion, cynicism, and profession efficacy) above thresholds. Where symptoms overlap, this indicates the proportion of respondents exceeding thresholds on both symptoms. Where all three symptoms overlap, this indicates the proportion of respondents exceeding thresholds on all three symptoms.

Of the 239 participants who completed all three MBI-GS subscales, 115 (48.1%) reported high levels of EE (≥3.2), 72 (30.2%) reported high levels of CY (≥2.6), and 39 (16.3%) reported low levels of PE (≤3.8). As shown in Figure 1, 138 respondents (57.7%) exceeded the threshold for high levels of burnout on at least one dimension, 71 (29.7%) exceeded the thresholds on at least two dimensions, and 17 (7.1%) exceeded the thresholds on all three.

### Differences Between Clinical and Non-clinical Staff

There were no significant differences in job satisfaction between clinical and non-clinical staff, $Welch^{\prime }s{\;t}_{\left(53.45\right)}$ = −0.057, *p* =0.955, *d* = −0.01, 95% CI [-0.35 – 0.33]. Clinical staff were found to be more emotionally exhausted than non-clinical staff with a medium effect size, *t*_(208)_ = 2.61, *p* =0.010, *d* = 0.45, 95% CI [0.11 – 0.79]. No differences were found for cynicism [*t*_(208)_ = 1.69, *p* = 0.094, *d* = *0.29*, 95% CI [-0.05, 0.62]] or professional efficacy [*t*_(208)_ = −1.95, *p* = 0.053, *d* = −0.333, 95% CI [−0.67, 0.004]].

### Associations Between Job Satisfaction and Symptoms of Burnout

Scores on the emotional exhaustion and cynicism subscales were highly correlated (*r* = 0.648, *p* < 0.0006), while scores on the professional efficacy subscale were inversely related to scores on the emotional exhaustion (*r* = −0.293, *p* < 0.0006) and cynicism (*r* = −0.453, *p* < 0.0006) subscales. Job satisfaction was negatively correlated with both emotional exhaustion (*r* = −0.483, *p* < 0.0006) and cynicism (*r* = −0.613, *p* < 0.0006), and positively correlated with professional efficacy (*r* = 0.443, *p* < 0.0006).

Multiple regression revealed the three symptoms of burnout together explained 42% of the variance in job satisfaction, adjusted *R*^2^ = 0.42, *F*_(3, 235)_ = 58.42, *p* < 0.001. Emotional exhaustion (β = −0.14, *p* = 0.009), cynicism (β = −0.34, *p* < 0.001), and professional efficacy (β = 0.26, *p* < 0.001) were all significant predictors of job satisfaction. Assumption checks indicated that multicollinearity was not a concern (Emotional exhaustion, Tolerance = 0.58, VIF = 1.72; Professional efficacy, Tolerance = 0.79, VIF = 1.26; Cynicism, Tolerance = 0.50, VIF = 1.98).

### Sources of Stress, Rated, and Ranked

The mean ratings for the 19 potential sources of stress are listed for clinical and non-clinical staff in Table 4 ranked from most to least common. The most common sources of stress for clinical staff were (1) heavy workload, (2) poor remuneration, (3) working long hours, (4) lack of recognition for the veterinary profession from the general public, and (5) client expectations. The most common sources of stress for non-clinical staff were (1) heavy workload, (2) admin requirements, (3) poor remuneration, (4) lack of resources, and (5) unclear roles and responsibilities.

**Table 4:** Potential sources of stress for clinical and non-clinical staff.

**Source of stress**	**Clinical**		**Non-clinical**	
	**Mean (SD)**	**Rank**	**Mean (SD)**	**Rank**
Heavy workload	3.58 (1.27)	1	3.02 (1.35)	1
Poor remuneration	3.20 (1.58)	2	2.51 (1.55)	3
Working long hours	3.15 (1.46)	3	2.19 (1.40)	7
Lack of recognition for the veterinary profession from the general public	2.94 (1.42)	4	1.65 (1.70)	14
Client expectations	2.78 (1.46)	5	2.05 (1.80)	11
Client emotions	2.70 (1.45)	6	2.26 (1.83)	6
Lack of resources	2.52 (1.29)	7	2.35 (1.33)	4
Ethical dilemmas	2.48 (1.28)	8	1.56 (1.28)	16
Client dissatisfaction with treatment outcomes	2.42 (1.37)	9	2.14 (1.74)	9
Inadequate time off	2.41 (1.77	10	1.77 (1.17)	13
Lack of support or respect from colleagues	2.41 (1.36)	11	2.16 (1.48)	8
Lack of respect from clients	2.39 (1.44)	12	1.91 (1.62)	12
Animal euthanasia	2.25 (1.25)	13	1.23 (1.34)	18
Occupational hazards	2.20 (1.24)	14	1.56 (1.08)	15
Unclear role and responsibilities	2.05 (1.24)	15	2.28 (1.16)	5
Admin requirements	2.05 (1.50)	16	2.58 (1.58)	2
Job insecurity	1.58 (1.01)	17	1.44 (1.08)	17
Non-paying clients	1.42 (1.41)	18	2.14 (1.95)	10
Time “on call”	1.14 (1.40)	19	1.0 (1.29)	19

### Associations Between Sources of Stress and Symptoms of Burnout and Job Satisfaction

Pearson's correlations between sources of stress and symptoms of burnout and job satisfaction for clinical and non-clinical staff are reported in Table 5.

**Table 5:** Correlations between sources of stress and symptoms of burnout and job satisfaction.

**Sources of stress**		**Emotional exhaustion**	**Cynicism**	**Professional efficacy**	**Job satisfaction**
Heavy workload					
	Clinical	0.590^*^	0.357^*^	−0.024	−0.248
	Non-clinical	0.488^*^	0.268	−0.135	−0.224
Poor remuneration					
	Clinical	0.373^*^	0.348^*^	−0.142	−0.361^*^
	Non-clinical	0.241	0.303	−0.152	−0.473
Working long hours					
	Clinical	0.524^*^	0.321^*^	−0.070	−0.204
	Non-clinical	0.447	0.239	−0.201	−0.193
Client expectations					
	Clinical	0.286^*^	0.080	0.021	−0.016
	Non-clinical	0.165	−0.067	0.233	0.166
Lack of recognition for the profession					
	Clinical	0.365^*^	0.212	−0.007	−0.110
	Non-clinical	0.159	0.244	0.135	−0.118
Client emotions					
	Clinical	0.303^*^	0.078	−0.021	−0.036
	Non-clinical	0.125	0.014	0.261	0.221
Lack of resources					
	Clinical	0.392^*^	0.303^*^	−0.076	−0.332^*^
	Non-clinical	0.241	0.242	−0.297	−0.214
Lack of support or respect from colleagues					
	Clinical	0.373^*^	0.389^*^	−0.148	−0.517^*^
	Non-clinical	0.280	0.727^*^	−0.489	−0.509^*^
Client dissatisfaction with treatment outcomes					
	Clinical	0.131	−0.021	0.079	0.070
	Non-clinical	0.167	−0.043	0.228	0.098
Ethical dilemmas					
	Clinical	0.289^*^	0.150	−0.123	−0.156
	Non-clinical	0.351	0.232	−0.040	−0.045
Lack respect from clients					
	Clinical	0.281^*^	0.124	0.084	−0.088
	Non-clinical	0.188	0.143	0.091	0.088
Inadequate time off					
	Clinical	0.507^*^	0.409^*^	−0.203	−0.303^*^
	Non-clinical	0.265	0.013	−0.017	−0.140
Admin requirements					
	Clinical	0.084	−0.026	−0.090	−0.014
	Non-clinical	0.464	0.412	−0.261	−0.229
Unclear about role and responsibilities					
	Clinical	0.262^*^	0.392^*^	−0.303^*^	−0.332^*^
	Non-clinical	0.189	0.463	−0.373	−0.408
Occupational hazards					
	Clinical	0.331^*^	0.239	−0.078	−0.157
	Non-clinical	0.275	0.362	−0.095	−0.320
Animal euthanasia					
	Clinical	0.229	0.083	0.015	−0.023
	Non-clinical	0.043	−0.048	0.220	0.178
Job insecurity					
	Clinical	0.195	0.269^*^	−0.225	−0.244
	Non-clinical	0.377	0.608^*^	−0.590^*^	−0.452
Non-paying clients					
	Clinical	0.062	−0.088	0.011	−0.060
	Non-clinical	0.195	0.063	0.070	0.095
Time “on call”					
	Clinical	0.185	−0.022	0.061	0.018
	Non-clinical	−0.020	0.170	0.011	−0.374

* *p < 0.0006*.

For clinical staff, emotional exhaustion was moderately associated (*r* ≥ 0.4) with heavy workload, working long hours, and inadequate time off, and weakly associated (*r* ≥ 0.2) with poor remuneration, client expectations, lack of recognition for the profession, client emotions, lack of resources, lack of support or respect from colleagues, ethical dilemmas, lack of respect from clients, unclear roles and responsibilities, occupational hazards, and animal euthanasia.

For non-clinical staff, emotional exhaustion was moderately associated (*r* ≥ 0.4) with heavy workload, working long hours, and admin requirements, and weakly associated (*r* ≥ 0.2) with poor remuneration, lack of resources, lack of support or respect from colleagues, ethical dilemmas, inadequate time off, occupational hazards, and job insecurity.

For clinical staff, cynicism was moderately associated (*r* ≥ 0.4) with inadequate time off, and weakly associated (*r* ≥ 0.2) with heavy workload, poor remuneration, working long hours, lack of recognition for the profession, lack of resources, lack of support or respect from colleagues, unclear roles and responsibilities, occupational hazards, and job insecurity.

For non-clinical staff, cynicism was strongly associated (*r* ≥ 0.6) with lack of support or respect from colleagues and job insecurity, moderately associated (*r* ≥ 0.4) with admin requirements and unclear roles and responsibilities, and weakly associated (*r* ≥ 0.2) with heavy workload, poor remuneration, working long hours, lack of recognition for the profession, lack of resources, ethical dilemmas, and occupational hazards.

For clinical staff, professional efficacy had a weak negative association (*r* ≥ −0.2) with inadequate time off, unclear roles and responsibilities, and job insecurity.

For non-clinical staff, professional efficacy had a moderate negative association (*r* ≥ −0.4) with lack of support or respect from colleagues and job insecurity, and a weak negative association (*r* ≥ −0.2) with working long hours, lack of resources, admin requirements, and unclear roles and responsibilities, and a weak positive association (*r* ≥ 0.2) with client expectations, client emotions, client dissatisfaction with treatment outcomes, and animal euthanasia.

For clinical staff, job satisfaction had a moderate negative association (*r* ≥ −0.4) with lack of support and respect from colleagues, and a weak negative association (*r* ≥ −0.2) with heavy workload, poor remuneration, working long hours, lack of resources, inadequate time off, unclear roles and responsibilities, and job insecurity.

For non-clinical staff, job satisfaction had a moderate negative association (*r* ≥ −0.4) with poor remuneration, lack of respect or support from colleagues, unclear roles and responsibilities, and job insecurity, a weak negative association (*r* ≥ −0.2) with heavy workload, lack of resources, admin requirements, occupational hazards, and time “on call,” and a weak positive association (*r* ≥ 0.2) with client emotions.

### Veterinary Care Workers' Perspectives on Factors Contributing to Stress and Wellbeing

Thematic analysis of open-ended survey responses and focus group transcripts revealed three primary sources of work-related wellbeing (people, practice, and purpose) and three primary sources of work-related stress (communication, clients, and case load; see Table 6 for summary).

**Table 6:** Summary of themes with examples.

**Sources of work-related wellbeing**			**Sources of work-related stress**		
**People**	**Practice**	**Purpose**	**Communication**	**Clients**	**Case load**
– Team members – Comradery – Supportive – Hardworking and dedicated – Shared interests and values	– Varied – Stimulating – Rewarding – Collaborative – Gold standard care – Outcomes	– Caring for animals – Supporting families – Making a difference to patients' and families' lives	– Between teams and departments – Between vet and nursing staff – Between management and those “on the floor” – Large teams – Politics	– High / unrealistic expectations – Distress and anger – Financial constraints – Lack of respect	– Staff to patient ratios – Long hours, no paid overtime – Case load in relation to complexity – Staff turnover, staff shortages

#### People

The majority of clinical and non-clinical participants identified their relationships with colleagues as a crucial source of wellbeing. Indeed, one participant reported that “*work is similar regardless of workplace… it's the people that make the difference*.” Many described an atmosphere of comradery and “*team spirit*” among peers who, for the most part, they found to be supportive and respectful:

“*I really appreciate how much of a family I consider the team*”

“*I feel we have a supportive team who are open to feedback and change*”

“*I feel heard*”

“*Everyone helps each other out and works as a team*”

Participants appreciated working alongside colleagues who they described as hardworking and dedicated teammates who were “*striving to be the best*” (resident / intern). One clinician reported they were grateful to work with “*highly intelligent, motivated, opinionated people*” with “*diverse skills and knowledge*.” Others felt connected to colleagues with shared interests and values (e.g., “*I enjoy being around other like-minded people who can support me and my decision-making*”).

#### Practice

Many participants reported feeling proud to work at a hospital that was delivering “*gold standard*” care with the best available facilities and technology (“*It helps me feel that patients are really getting the best treatment*”). Clinical staff in particular described their work as intellectually stimulating and rewarding, and they appreciated being able to collaborate with and learn from highly experienced colleagues (e.g., “*I like the multidisciplinary approach, and that I learn something new every day*”).

#### Purpose

Many participants reported that caring for animals and supporting families were the most rewarding parts of their job. They described feeling fortunate to interact daily with “*beautifully natured animals*” and to work in an environment where others shared their passion. Both clinical and non-clinical staff described the satisfaction they often felt at being able to make a difference to patients' and families' lives:

“*Ultimately, working to save patient lives is what I enjoy doing. Working in a critical care and emergency setting, everything you do matters, and knowing that I make a difference to a patient and their family's lives*”

“*Feeling that at times my contribution can help a family through a hard time*”

“*The feeling of contributing to something of great importance (helping families and pets in hard times). This part of my job fills me with pride*”

#### Communication

Poor communication—both within and between teams and departments—was commonly cited as a source of stress with many participants describing issues with “*politics*” and “*hierarchy*.” Frictions between management and those on the “*floor*” or between clinical and non-clinical staff were particularly prevalent throughout responses:

“*The ‘behind the scenes' people get forgotten*.”

“*Non-clinical staff members making decisions on clinical processes based on how they feel about a situation is stressful, as they have no experience or knowledge to make that decision that hugely impacts the team*”

“*The feeling that sometimes the higher-ups don't care about us*”

Lack of clarity around roles and responsibilities was also a source of frustration for many, with one participant reporting “*not always knowing exactly what I need to do*.” Similarly, another participant described “*the lack of clear managerial structure, the lack of accountability each person has to do their job, the lack of transparency around what exactly each person's role is… the lack of cooperation across department*.”

Some participant attributed the communication problems to working in large teams and in a growing organization (e.g., “*A large team means that at times, it's difficult to set standards, expectations and clear pathways which can be challenging/stressful*”) with one participant described feeling as though they were “*getting lost in the system*.”

#### Clients

Managing client emotions was a key source of stress for both clinical and non-clinical staff. They reported that “*the emotional side of sick animals*” was taxing and that managing client expectations about treatment options could be challenging (e.g., “*People expect their dog to come in broken and to leave fixed and that's not always what happens, and you can see anger from it*”). One participant reported feeling exhausted by the “*constant emotional unloading owners do. Sometimes I feel like I use up all my empathy on people I don't know very well, and I have none left for my family at the end of the week*.” Another participant described their work as “*30% medicine, 30% admin, and then 30% being a therapist*.”

Several clinical staff explained that they considered themselves “*animal people*” rather than “*people people*” and, as a result, they found the human side of their work particularly challenging (e.g., “*We go into this industry because we care about animals, not so much people, so dealing with people can be stressful*”). On the other hand, those working in client services felt more confident in their ability to negotiate with owners (e.g., “*We've chosen to do the human side so we've got an understanding that people can be [rude] sometimes and it's not personal*”).

Those who had worked in the industry for many years noted cultural changes in the relationships people have with their pets had intensified the emotional nature of veterinary care (e.g., “*Animals are becoming more and more the children of people… They will say* ‘*you are killing my animal*”').

A number of participants reported that anger and aggression were particularly difficult emotions to deal with and that “*talking to clients about finances*” could be uncomfortable.

“*I got berated by this client and now every time I see that client in my schedule, I don't sleep the night before because it creates that much stress and anxiety in me having this angry client that comes in on a regular basis*.”

“*The ongoing price rises make me feel like I only get to help the wealthy dogs. I feel terrible when owners come in who are young and not financially secure or owners have other financial difficulties, like losing a job or caring for a partner, and they're dedicated and sensible, but we just cost too much*”

#### Case Load

The most commonly reported source of stress across all participants was workload. Many described working long hours with few breaks, being overwhelmed by inadequate staff-to-patient ratios, and feeling that they were not fairly remunerated (i.e., low salaries and no paid overtime). One participant described “*always being pushed to the limit physically and mentally*,” while a specialist vet reported there was an expectation “*to do more than you are able in a day”*. Nursing teams were reportedly worst affected by issues of staff shortages and turnover (“*Nurse to patient ratio is sometimes ridiculous*,” “*The ratio of nurses to patients is unsafe at times and too many people are doing dangerous overtime*”), which had been exacerbated by the Covid-19 pandemic (“*There is just no slack in the system at the moment*”). A number of staff expressed concern that profit was at times prioritized over staff and patient welfare, and that the quality of their work was compromised by unreasonable caseloads (“*No one in leadership makes the call to turn patients away or come in to help*”).

## Discussion

The aim of this study was 2-fold: (1) to identify factors that are associated with burnout and job satisfaction among clinical and non-clinical veterinary care workers, and (2) to explore factors that exacerbate stress and promote wellbeing at work.

### Prevalence of Burnout and Job Satisfaction

Previous research into veterinary carer worker burnout in Australia, conducted using a variety of burnout measures, has reported that the prevalence of high levels of burnout ranges from 24 to 53% (4–7). In the current study, using the MBI-GS, 57.7% of respondents exceeded the burnout threshold on at least one dimension. Almost half of the respondents (48.1%) reported high levels of emotional exhaustion, while 30.2% reported high levels of cynicism, and 16.3% reported low levels of professional efficacy.

High levels of emotional exhaustion have previously been reported in international studies of veterinary burnout using the MBI-GS. For example, 46.2% in Canadian vets (47), 27% in Dutch vets (48), and 58.3% in North American veterinary technicians (32). However, unlike our study, these studies only included clinical workers, and we found clinical workers were significantly more emotionally exhausted than their non-clinical colleagues. Previous research including clinical as well as non-clinical veterinary staff has reported considerably lower rates of burnout than studies including clinical samples only (2).

Despite the prevalence of burnout symptoms in the current study, four out of five participants (80.7%) reported being satisfied with their job. This finding—that high levels of burnout in veterinary care workers is not necessarily associated with low levels of job satisfaction—is consistent previous research conducted in Canada where 83% of clinical and non-clinical workers were satisfied with their job despite one-in five experiencing high levels of burnout (2).

### Sources of Stress

For both clinical and non-clinical staff, heavy workload was the most commonly reported source of stress. This result was echoed in focus groups where both clinical and non-clinical staff reported caseload to be a significant source of stress. The negative impact of workload on stress in the veterinary industry is well-established with several studies in Australia and overseas reporting workload to be a primary source of stress (33–36, 38, 40).

Other common sources of stress for clinical workers (in order of prevalence) included poor remuneration, working long hours, lack of recognition for the profession, client expectations, and client emotions. For non-clinical workers, other common sources of stress (in order of prevalence) included admin requirements, poor remuneration, lack of resources, unclear roles and responsibilities, and client emotions. In addition to heavy workload, therefore, poor remuneration and client emotions were common sources of stress across the organization.

For clinical staff, nearly all sources of stress were associated with emotional exhaustion and cynicism, indicating that clinical staff are overwhelmed. For non-clinical staff, emotional exhaustion was only associated with heavy workload and cynicism was strongly associated with lack of respect or support from colleagues and job insecurity. The issue of communication with colleagues and not feeling valued for one's role in the organization was reiterated in focus groups.

Professional efficacy was generally high across the organization, which is consistent with previous research in veterinary settings (2, 32, 47). Indeed, in the present study the most common sources of stress were not associated with professional efficacy for clinical or non-clinical staff.

However, all of these associations should be interpreted with caution since correlation does not imply causation. For example, the data revealed a weak but positive association between client emotions and professional efficacy as well as client emotions and job satisfaction for non-clinical staff. This association likely reflects the heterogeneity of the non-clinical sample which includes many groups who do not have client-facing roles. Hence, it is possible that those non-clinical staff who have higher professional efficacy and job satisfaction also coincidentally have more contact with clients and higher exposure to client emotions. Indeed, the focus groups revealed that client emotions were a common source of stress for those clinical and non-clinical staff who are client-facing.

### Sources of Wellbeing

Data synthesized from focus group transcripts and open-ended survey responses indicated that the main sources of wellbeing across the organization included people, practice, and purpose. Although these themes were found among both clinical and non-clinical staff, practice and purpose were particularly relevant for clinical staff. This is consistent with prior research which found using their specialized skills and knowledge to make a difference buffered animal care workers against all three components of burnout (32). Wallace (49) found meaningful work is significantly related to veterinarians' wellbeing. Specifically, veterinarians derive meaning from their work by helping animals and people, experiencing self-actualisation (i.e., by achieving complex challenges that required them to use their specialized skills and knowledge), and feeling as though they belong to the profession. In this study, non-clinical staff (particularly those not client facing) indicated they felt their work was less valued. However, a sense of connectedness with the team (i.e., people) was particularly important for non-clinical staff wellbeing. Indeed prior research found veterinary team culture can have significant influence on individual levels of burnout and job satisfaction (2).

### Implications

Our study findings underscore the potential wellbeing benefits of (1) managing staff workload, (2) improving communication between teams, and (3) helping clinical and non-clinical staff to manage or cope with emotionally challenging client encounters for the animal hospital under evaluation.

### Managing Workload

Both clinical and non-clinical participants in the present survey reported heavy workload as the most prevalent source of stress at work. Consistent with Wallace (49) we found that clinical staff with heavier workload reported poorer wellbeing (higher emotional exhaustion) (49). Workload is determined in part by client demand, in part by organizational expectations, by scheduling processes, and by the availability of staff and resources to cope with demand. There is no doubt that veterinary hospital staff workload has been immeasurably impacted by the COVID-19 pandemic, which prevented veterinary hospitals in Australia from employing essential clinical workers from overseas (there is a shortage of veterinary specialists and technicians or nurses in Australia).

In this macroeconomic context, it may not be feasible to modify clinicians' workload without negatively impacting patient care (and in turn, clinicians' experience of purpose and meaning). Research suggests, however, that workload in and of itself is not necessarily sufficient to induce emotional exhaustion in veterinary care workers: an important determinant of the impact of workload on stress and burnout may be autonomy and control over work time and length of shifts (32). Similarly, in the context of human healthcare, work hours and caseload predict global burnout only indirectly, *via* their effects on either perceived workload or autonomy (50). These findings suggest that interventions designed to reduce burnout by limiting work hours may be more effective if healthcare providers are also given some autonomy or choice over their workload and hours. Indeed work autonomy (i.e., the freedom to determine how to perform one's job) is associated with lower levels of burnout among human healthcare providers (51). Hence, an organization may mitigate the stress associated with heavy workload by providing staff (clinical or non-clinical) with a degree of autonomy or choice over their work schedule: when they work, if not how long or how often they work.

### Managing Client Emotions and Expectations

Consistent with previous research conducted in veterinary care settings (36, 41), managing client expectations and client emotions were prominent sources of stress reported by both clinical and non-clinical staff in open text survey responses and focus groups, and were rated among the most prevalent sources of stress by both groups. For instance, Wallace (49) found that veterinarians who frequently have difficult interactions with clients find their work is less meaningful (49). It has been suggested that the emotional demands experienced by veterinarians may be even greater than that experienced by their human counterparts because their work involves caring for both humans and animals (52). These dual-caring roles are often emotionally intense and may make veterinary care providers more prone to burnout.

It may be difficult to modify the emotional demands associated with managing client expectations and responding to client emotions. However, in a recent study of burnout symptoms in human healthcare workers (in pain management), we found that clinicians' confidence in their ability to identify and respond to patient emotions was associated with a higher sense of personal accomplishment, and clinicians' confidence in their ability to identify and respond to their own emotions was associated with lower levels of emotional exhaustion and depersonalization (53). These findings suggest that interventions aimed at improving veterinary care workers' ability and confidence to manage client emotions and their own emotions may protect against developing symptoms of burnout.

Managers may also consider exercising care during the hiring and onboarding process to ensure that prospective employees have the skills and resources to cope with the demands of client expectations and emotions. Training might include workshops or video recorded modules to calibrate employee expectations of client emotions and behavior, to provide employees with strategies for responding to client emotions during difficult interactions, and skills or strategies for managing their own residual discomfort in a constructive manner in the wake of difficult client interactions (e.g., mindfulness, relaxation strategies, self-compassion, re-appraisal, and low-impact peer-debriefing).

### Communication

Consistent with the present research, previous studies have found that workplace relationships are among the primary reasons for leaving the veterinary profession (13), and at the heart of all relationships is communication. In the current study, open text survey responses, focus groups, and ratings of common sources of stress revealed a number of sources of stress related to communication, feeling disrespected or not valued by colleagues, unclear roles and responsibilities, internal politics and lack of certainty around protocols and procedures. Communicating across organizational levels and teams can be challenging in large organizations, particularly during a time of rapid change (as was experienced during the COVID-19 pandemic). During time of crisis, changes occur rapidly and unexpectedly as organizations make adaptations to macroeconomic and environmental demands, and communication gaps or delays are almost inevitable. In such circumstances we recommend leaning on (or developing) a strong culture of social support, cohesion, and trust to give employees the confidence to ask for help when they are unsure of role responsibilities, or procedures—without fear of reputational risk. In order to build a culture of mutual support and trust, it may be important to encourage team leaders to take deliberate steps to express appreciation for the work of team members as this is not something they would necessarily get from management or from clients directly.

### Summary of Recommendations

The purpose of identifying sources of stress and wellbeing in the current specialist animal hospital setting was to develop an evidence base on which to make recommendations for targeted interventions to support the wellbeing of staff in the specialist animal hospitals under examination, and specialist animal hospitals more generally. The results of the current study suggest that the following strategies have the potential to improve staff wellbeing and reduce stress in the specialist animal hospital under consideration, and specialist animal hospitals more broadly:

1. Look for ways to provide staff (clinical or non-clinical) with a degree of autonomy or choice over their work schedule
2. Provide staff with skills for responding to client emotions
3. Provide staff with skills for managing their own residual discomfort in response to client emotions
4. Develop a practice (and culture) of appreciation: recognizing demonstrations of skill, effort, cooperation, or patient-centredness.

### Strengths and Limitations

This is the first study in Australia to examine the prevalence and predictors burnout and job satisfaction in a specialist small animal veterinary hospital, where an interdisciplinary team of veterinarians, nurses/technicians, and support staff work collaboratively. The current study used a mixed-methods approach as a means of cross-checking the validity of survey results and identifying source of stress and wellbeing that may not have been identified in previous research. Using this approach, we identified not only a number of factors that are consistent with previous research (e.g., heavy workload, poor remuneration, working long hours), but our open-text response and focus group data also revealed novel sources of stress and wellbeing in the veterinary care industry, specifically communication with colleagues, management, and clients as a source of stress and practice and purpose as sources of wellbeing.

As the design of this study was cross-sectional, associations between variables should be interpreted with caution. Factors that are predictive of burnout are not necessarily causative. Furthermore, it should be noted that factors contributing to burnout in organizations are dynamic. Hence, the results of the current study only reflect associations that were present at a particular point in time, and we should be mindful that these associations are not necessarily stable. Relatedly, it is important to recognize that the research was undertaken during the Covid-19 pandemic when the veterinary health care system was under enormous stress due to staffing shortages and a boom in pet ownership (54), not to mention the other pandemic-related stressors affecting staff during this time. It is therefore likely that rates of burnout, particularly emotional exhaustion, were elevated during the study period. Moreover, after the implementation of strategies to address sources of stress, we would expect to see improvements in staff wellbeing (although this is outside the scope of the present study). Future research is recommended to evaluate the impact of these suggested interventions on burnout in specialist animal hospitals.

Factors associated with stress and burnout in veterinary hospitals may be idiosyncratic to the organizational setting and culture. At the same time, the results of the current study are consistent with previous research conducted in veterinary care settings, indicating that there may be sources of stress and wellbeing that are common across specialist animal hospitals.

## Conclusion

Despite job satisfaction being relatively high in veterinary care organizations, levels of emotional exhaustion and cynicism may also be relatively high. The current research identified communication, caseload, and client emotions as factors which may be contributing to stress, while people, purpose, and practice (i.e., varied and challenging work) were a consistent source of wellbeing across clinical as well as non-clinical staff. The results of the current research are consistent with findings both nationally and internationally suggesting that interventions to address burnout symptoms in veterinary care providers may benefit from alleviating these stress factors and taking steps to enhance these wellbeing factors.

## Data Availability Statement

The raw data supporting the conclusions of this article will be made available by the authors, without undue reservation.

## Ethics Statement

The studies involving human participants were reviewed and approved by Human Research Ethics Committee of the Northern Sydney Local Health District. The patients/participants provided their written informed consent to participate in this study. Written informed consent was obtained from the individual(s) for the publication of any potentially identifiable images or data included in this article.

## Author Contributions

CA-J and AM contributed to conception, design of the study, and analyzed the data. CA-J managed the data collection, facilitated the focus groups, and drafted the abstract, introduction, and discussion. AM drafted the methods and results. Both authors contributed to manuscript revision, read, and approved the submitted version.

## Funding

This research was funded by a donation to Pain Foundation Limited by the Small Animal Specialist Hospital (SASH), Sydney, Australia.

## Conflict of Interest

CA-J receives financial reimbursements for conducting communication workshops in the human and animal healthcare industries. The remaining author declares that the research was conducted in the absence of any commercial or financial relationships that could be construed as a potential conflict of interest.

## Publisher's Note

All claims expressed in this article are solely those of the authors and do not necessarily represent those of their affiliated organizations, or those of the publisher, the editors and the reviewers. Any product that may be evaluated in this article, or claim that may be made by its manufacturer, is not guaranteed or endorsed by the publisher.
